# Health responses to a new high-voltage power line route: design of a quasi-experimental prospective field study in the Netherlands

**DOI:** 10.1186/1471-2458-14-237

**Published:** 2014-03-07

**Authors:** Jarry T Porsius, Liesbeth Claassen, Tjabe Smid, Fred Woudenberg, Danielle RM Timmermans

**Affiliations:** 1 Department of Public and Occupational Health, EMGO Institute for Health and Care Research, VU University Medical Center, P.O. Box 7057, Amsterdam, MB 1007, The Netherlands; 2 KLM Health Services, P.O. Box 7700 (SPL/AG), Schiphol, ZL 1117, The Netherlands; 3 Municipal Health Service, P.O. Box 2200, Amsterdam, CE 1000, The Netherlands; 4 National Institute for Public Health and the Environment, P.O. Box 13720, Bilthoven, BA, The Netherlands

**Keywords:** Power lines, Health complaints, Symptom reports, Environmental concerns, Anxiety, Environmental incidents, Modern health worries, Environmental risk perception, Nocebo, Attribution

## Abstract

**Background:**

New high-voltage power transmission lines will be introduced due to increasing demand for reliable and renewable energy supplies. Some residents associate non-specific health complaints with exposure to electromagnetic fields from nearby power lines. This study protocol describes the design and rationale of a prospective study investigating whether the introduction of a new power line triggers health responses in residents living nearby.

**Methods/Design:**

The study is designed as a quasi-experimental field study with two pretests during the construction of a new power line route, and two posttests after it has been put into operation. Key outcomes are self-reported non-specific somatic and cognitive health complaints, and attribution of these health complaints to a power line. The main determinant is proximity to the new power line route. One member of every household (n = 2379) residing in close proximity (0-500 meters) to the overhead parts of a new power line route in the Netherlands is invited to participate, as well as a sample of household members (n = 2382) residing farther away (500-2000 meters). Multilevel analysis will be employed to test whether an increase in key outcome measures is related to proximity to the line. Longitudinal structural equation models will be applied to test to what extent health responses are mediated by psychosocial health mechanisms and moderated by negative oriented personality traits.

**Discussion:**

This is the first study to investigate health responses to a new power line route in a prospective manner. The results will provide theoretical insight into psychosocial mechanisms operating during the introduction of an environmental health risk, and may offer suggestions to policymakers and other stakeholders for minimizing adverse health responses when introducing new high-voltage power lines.

## Background

New high-voltage power lines are being introduced into the environment as a result of increasing demand for reliable and renewable energy supplies [1]. In the Netherlands, a total of 350 kilometers of 380 kV overhead transmission lines will be introduced in the near future. The introduction of such a new power line route may have a considerable impact on residents living nearby. In addition to burdens such as visual intrusion (for overhead power lines), noise and a reduction of property values (see [2,3] for a full overview), potential health risks of exposure to extremely low frequent electromagnetic fields (ELF-EMF) emitted by power lines can be perceived as a burden as well.

Claims about health effects from exposure to ELF-EMF have been made since the late 70s [4]. Pooled analyses [5,6] showed a small but consistent association between childhood leukemia and living near an overhead power line, and led to renewed attention for the potential health risks of power lines. A recent report by the World Health Organization [7] concludes that when it comes to the link between ELF-EMF from power lines and childhood leukemia “…on balance, the evidence is not strong enough to be considered causal, but sufficiently strong to remain a concern” (p. 12). The scientific evidence for other health issues investigated (e.g. other types of cancer and neurological disease) is weaker and in some cases “sufficient to give confidence that magnetic fields do not cause the disease” (p. 12). Despite, or because of the inconclusive evidence, residents who live in the proximity of an overhead power line are more concerned about the health risks than residents living farther away [8]. Interviews with residents living in close proximity to overhead power lines reveal that they associate non-specific health complaints such as tiredness, headaches and neurological problems with exposure to ELF-EMF from nearby power lines [9]. Moreover, these health risks seem more important to them than other burdens such as the aesthetic impact [10,11].

From risk perception research it is well known that risk perceptions are determined by direct or indirect communication about a potential threat through formal and informal social networks [12]. Media reports on the health effects of EMF exposure do not reflect the current scientific state of evidence [13-15]. Together with information from other sources (e.g. peers, government, opinion leaders, one’s own experience, etc.) risk perceptions of EMF from power lines might become amplified. Recent studies show that power lines are perceived by the general public to be one of the riskiest sources emitting EMF [16,17]. An electric blanket, for example, produces electric fields comparable to power lines, but these fields are perceived as less dangerous [18]. People’s judgments about danger involve perceptions of severity and immediacy, controllability, voluntariness, equity issues, as well as the degree to which a risk is known to science [19,20]. These factors might in part explain the relatively high perceived risks of EMF emitted by power lines when compared to other EMF sources. In addition to these cognitive evaluations of a risk, affective evaluations are important in judging whether a situation is hazardous [21-23]. A recent study showed that people implicitly associate power lines with being unhealthy [24], suggesting a spontaneous negative evaluation of the health risks of power lines. Moreover, residents living in proximity to a power line relate this device to an uncomfortable feeling [25]. Thus, the perceived risks of power lines are relatively high, both on an affective and cognitive level.

The relatively high perceived risks of power lines may adversely affect well-being and health of residents living near a power line through a psychosocial pathway linking exposure to a potential environmental hazard to symptom reporting [26-28]. For equipment emitting EMF, such as power lines, key elements in this psychosocial pathway are the subjective perception of being exposed and perceiving this exposure as a health risk [29]. Distance to a risk object [30-32], visibility of the object [33,34], and information received about the exposure [35,36] might be important determinants of feeling exposed. Effects of perceived exposure on health were found in experimental studies, demonstrating that symptom reporting increased in healthy participants after being exposed to sham EMF, supposedly emitted by visibly present electrical equipment [37,38]. Similar effects were also found in EMF provocation studies with patients who describe themselves as sensitive to EMF [39-41]. These findings are interpreted as nocebo effects [42]; the phenomenon whereby expectation of a negative outcome leads to worsening of a symptom [43]. Little is known about the mediating mechanisms in nocebo responses. Symptom perception models stress the role of negative affect (e.g. anxiety) in the experience of non-specific symptoms [44]. For example, complex biochemical mechanisms linking anxiety to pain may explain nocebo-induced pain [45]. In addition to negative affect, a consistent association has been found between worrying about health effects of environmental exposures and reporting non-specific health complaints [46-49].

Another important element in the psychosocial pathway is the role of symptom interpretation. It is well known that causal beliefs about one’s health complaints affect the experience of these symptoms [50-52]. For example, in a prospective study with neurology out-patients with unexplained symptoms, not attributing symptoms to psychological causes predicted poor health outcome [53]. Between 1.5 and 10% of the general population reports non-specific health complaints they attribute to EMF exposure from electrical equipment [42]. How causal beliefs about environmental exposures are linked to perceived health is not clear. According to Leventhal’s common sense model, people act as common-sense scientists when assessing the danger of possible illness threats. The stronger the symptom experience, the more people are in need of finding an explanation [54]. A recent study suggested that the attribution of symptoms to EMF is stronger when people report more intense symptoms [55]. Interviews with self-diagnosed electro-hypersensitive patients indicated that their attribution of symptoms to EMF began with a period of suffering from non-specific health complaints [56]. Subsequently, remarks by peers, information on the internet or television/newspaper coverage directed their attention to EMF as a cause of their symptoms. Case reports of patients illustrated that reported symptoms worsened when people became more convinced that they were suffering from electro-hypersensitivity, suggesting a reciprocal relationship between symptom intensity and the strength of causal beliefs regarding these symptoms [57]. An experimental study showed that non-specific symptoms attributed to perceived environmental exposure were experienced as more intense when at the same time bodily arousal was high due to other unrelated causes [58]. This dynamic relationship between symptom intensity and causal beliefs involving environmental exposures demonstrates the importance of both processes in understanding health responses to environmental risks.

In addition to psychosocial health mechanisms evoked by external cues, person-related factors are also part of the psychosocial pathway linking a potential hazard to symptom reporting [27,59]. Differences in personality might be associated with differences in risk perception and people’s interpretation of their symptoms, as well as the extent to which they experience and report these symptoms. In particular, negative oriented personality (NOP) traits such as neuroticism, trait anxiety or trait negative affect are related to symptom reporting [60]. However, evidence of the relationship of these traits with environmental risk perception [61-64] or symptom attribution to environmental factors [65-67] is mixed. NOP traits might also affect health by moderating nocebo effects. For example, in a range of placebo and nocebo experiments [68-70] health responses were shown to be moderated by dispositional pessimism, a NOP trait. This is also in line with a field study showing that the relationship between perceived noise from wind turbines and symptom reporting was moderated by NOP traits [71]. An explanation for these findings may be found in the perseverative cognition hypothesis, which states that health consequences of a stressor only occur after chronic activation of the cognitive representation of the stressor [72]. With respect to nocebo responses, negative expectations of an environmental exposure (or treatment) might only affect health when these cognitions are repeatedly activated [73]. Because individuals with a negative oriented personality tend to ruminate more [74-76], they might also be more susceptible to repeated activation of negative health expectations regarding environmental exposures.

**Figure 1:**
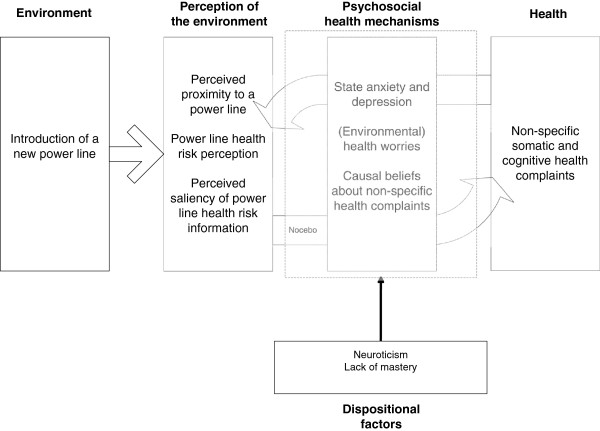
Conceptual framework for health responses after the introduction of a new power line.

We depict the key concepts for predicting health responses after the introduction of a new power line in a conceptual framework (Figure 1). In a large cross-sectional general population study [77], Baliatsas and colleagues found an association between perceived proximity to a power line and non-specific health complaints. However, they did not find an effect of actual distance to the nearest overhead power line. This suggests that the perception of living close to a power line is more important for developing health complaints than actual proximity. In a smaller cross-sectional study with residents actually living close to a power line, McMahan and Meyer [10] did not find a difference between living very close (on the easement) or less close (one block away) to a power line with regard to reporting non-specific symptoms such as headaches or difficulties in concentrating. They found that the level of worry about the presence of a power line was related to reporting health complaints only for those residents who lived very close to the power line. Neither study assessed causal beliefs. Moreover, the cross-sectional nature of these studies does not make it possible to draw conclusions about causality.

The main aim of the current study is to determine to what extent health responses to the introduction of a new power line occur. To our knowledge, this will be the first prospective study of health responses to a power line. The secondary aim consists of identifying mediating and moderating mechanisms explaining these health responses. The research questions are:

1. Do non-specific health complaints increase for residents living near a new power line after it has been put into operation, compared to residents living farther away?

2. Do causal beliefs about non-specific health complaints involving power lines increase for residents living near a new power line after it has been put into operation, compared to residents living farther away?

3. To what extent are health responses to a new power line route mediated by psychosocial health mechanisms and moderated by negative oriented personality traits?

This paper describes and discusses the design of a quasi-experimental prospective field study of health responses to the introduction of a new power line route in the Netherlands.

## Methods and design

### Study design

This study is designed as a quasi-experimental field study with two pretests during the construction of a new power line route and two posttests after it has been put into operation. Residents living nearby are compared to a non-equivalent control group of residents living farther away. The Medical Ethics Committee of the VU University Medical Center Amsterdam approved the protocol in 2012.

### Setting

**Figure 2:**
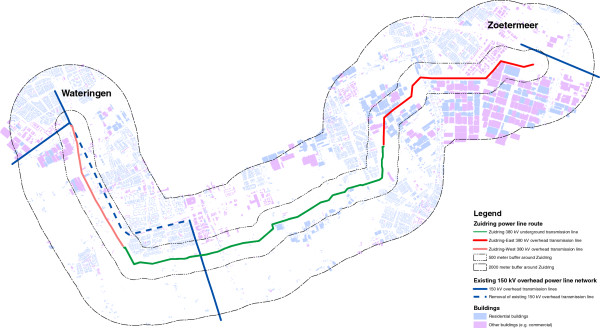
Geographical map of the study area.

**Figure 3:**
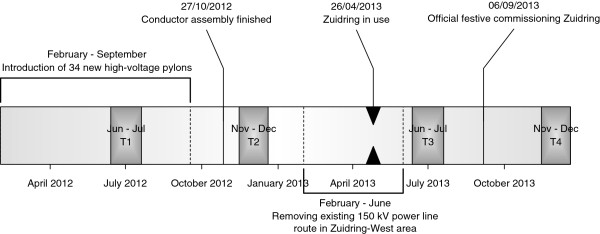
Timeline construction Zuidring power line route and timing of data collection.

New 380 kV overhead and underground power lines will be introduced in several parts of the Netherlands in the near future. The first construction of such a high-voltage power line route is the 22-kilometer Zuidring from the municipalities of Wateringen to Zoetermeer. The plan to build a new power line between these two cities was first publicly announced in 2006. After an environmental impact assessment of all possible routes and a procedure to involve all stakeholders, a definitive route was chosen by the national government in 2009. During the environmental planning process, residents were informed through local newspapers and leaflets distributed by national grid operator TenneT. Public meetings were held in order to engage residents in the planning process and answer questions from residents about routes and potential health effects of EMF. The Zuidring consists of three parts: 4.4 kilometers of overhead transmission lines from Wateringen till Delft (Zuidring-West), continuing with 10.7 kilometers underground till Pijnacker-Nootdorp (Zuidring-Underground) and ending with 6.8 kilometers of overhead transmission lines in Lansingerland (Zuidring-East, see Figure 2 for a geographical map of the area). For the overhead transmission lines, TenneT developed new so-called Wintrack pylons aimed at reducing the magnetic field zone with a minimal design promoting an unobtrusive presence in the landscape. The underground transmission line is the first 380 kV power line to be laid underground in the Netherlands. Along with the building of the Zuidring, parts of an existing 150 kV overhead transmission line in the Zuidring-West area were removed. See Figure 3 for an overview of important events in 2012 and 2013 regarding the construction of the Zuidring.

### Sampling method

**Figure 4:**
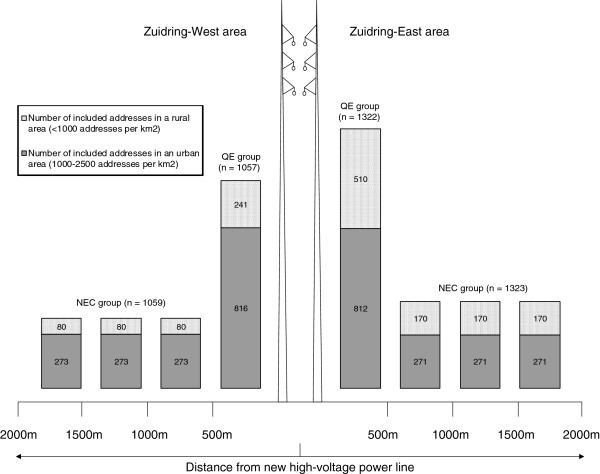
Random stratified sampling strategy for inclusion of household addresses.

Households were included based on proximity to the overhead parts of the Zuidring. We obtained addresses of households and geographical coordinates from the publicly accessible national building registry. Distances to the nearest overhead and underground parts of the Zuidring were calculated using ArcGIS 9.3.1 software and geographical information about the new power line route was provided by TenneT. See Figure 4 for an overview of our inclusion sampling strategy. Because previous research indicated that residents living within 500 meters of an existing overhead power line are more concerned about the health risks than residents living farther away [8], we included all available household addresses within 500 meters of the overhead parts of the Zuidring as our quasi-experimental (QE) group (n = 2379). For selecting our non-equivalent control (NEC) group, 55938 addresses were available residing between 500 – 2000 meters from the overhead parts of the Zuidring. Analysis based on available information regarding the density of addresses on postal code level revealed that households farther away from the Zuidring reside in a more urban area. To reduce potential confounding on health responses, we matched our control group to this environmental characteristic by means of random stratified sampling. First, information about degree of urbanization on postal code level was combined with all available household addresses. Since no households in our QE group resided in a very urban area (more than 2500 addresses per km^2^), all household addresses residing in this highest level of urbanization were excluded from the NEC group sampling frame (n = 24084). Then all remaining addresses (n = 31854) were stratified for area (Zuidring-West and Zuidring-East), distance category (500-1000 m, 1000-1500 m and 1500-2000 m) and degree of urbanization (less than 1000 addresses per km^2^ and 1000-2500 addresses per km^2^). We drew random samples from these strata matching the proportion of addresses in rural and urban areas of the QE group. As a result, 2382 addresses located between 500-2000 meters of the overhead parts of the Zuidring were included as our NEC group.

Additionally, we included all households residing within 500 meters of the 380 kV underground transmission line, excluding households within 2000 meters of the overhead parts of the Zuidring (complete underground sample, n = 502). To our knowledge there are no studies on the perceived health risks of underground 380 kV transmission lines, therefore we included this group for exploratory purposes.

Informed consent is obtained from participants via internet before filling in the digital questionnaire. However, according to the Medical Ethics Committee of the VU University Medical Center, there is no legal requirement to get informed consent for the present study.

### Power analysis

With an expected response rate of 30% and an attrition rate of 30% at every subsequent wave, we would end up with a total sample of 490 participants in our last wave. Power sensitivity calculations for a two-group pretest-posttest controlled design with a change score model were performed (for calculation method see [78]), given a significance level of 0.05 and a power of 0.80. Under these conditions the effect size of the minimal detectable difference between the two groups is 0.17 for the primary outcome measures (i.e. non-specific somatic and cognitive health complaints and causal beliefs involving power lines). This effect size may be categorized as small [79].

### Procedure

Our study will be performed in agreement with local municipal health services. All included households receive a postal letter with a request for one household member older than 18 to participate in the study. In this letter the study is presented as a longitudinal environmental health study aiming to relate changes in the environment to changes in health. To reduce response bias and demand characteristics, power lines are not mentioned. The letter contains a hyperlink to a digital questionnaire and a personal login and password. Invitations for follow-up questionnaires will be sent by email, with a maximum of three reminders. In the case of an invalid e-mail address, an invitation will be sent by postal letter. On request, residents are also able to participate in our study by receiving paper versions of the questionnaires.

All questions are asked in a fixed order starting with demographics, followed by questions pertaining to health, and closing with all environmental perception questions. See Figure 3 for a timeline depicting the exact timing of the four measurements (T1-T4). At each measurement wave, 50 euro gift certificates are randomly awarded to ten participants who filled out a questionnaire.

### Measures

#### Outcome measures

Table 1 shows the outcome measures and the timing of these measures in the study. Outcome measures are classified according to the layout of our conceptual framework (see Figure 1).

**Table 1:** Timing of measurements and used instruments

**Variables**	**Instruments**	**Questionnaires**			
		**T1**	**T2**	**T3**	**T4**
**Health**					
Non-specific somatic complaints	4DSQ-somatisation scale	X	X	X	X
Non-specific cognitive complaints	MOS-cognitive functioning	X	X	X	X
General health perception	1-item from SF-12 and 1-item from EQ5-D	X	X	X	X
Perceived change in health				X	X
**Psychosocial health mechanisms**					
Causal beliefs about health complaints	IPQ-cause scale (adapted)	X	X	X	X
Anxiety and depression	HADS	X	X	X	X
General health concerns	GHPQ (health concern scale)	X	X	X	X
Environmental health concern	MHW (adapted)		X	X	X
**Perception of the environment**					
Negative general health expectations of environmental factors			X	X	X
Negative personal health expectations of environmental factors		X	X	X	X
Perceived proximity to environmental risk objects		X	X	X	X
Perceived saliency of environmental health risk information		X	X	X	X
Perceived environmental change				X	X
**Dispositional factors**					
Demographics		X			
Neuroticism	NEO-FFI		X		
Lack of mastery	5-item mastery scale		X		

### Health

#### Non-specific somatic complaints

To measure non-specific somatic health complaints, we use the somatisation scale of the Dutch 4DSQ [80]. This scale consists of 16 non-specific somatic symptoms commonly reported in general practitioner practices, such as headaches, dizziness, low back pain. For each health complaint, participants indicate whether they were bothered by it during the previous week on a 5-point scale (ranging from no, through to constantly). Three symptoms were added to the list due to specific health expectations people may have of power lines, namely sleep problems, fatigue and tinnitus.

#### Non-specific cognitive complaints

Residents may also expect cognitive complaints from power lines in their vicinity [9]. The perception of cognitive functioning is assessed using a Dutch translation [81] of the MOS Cognitive Functioning Scale [82]. The scale consists of 6 items tapping the domain of general cognitive functioning (e.g. forgetfulness, difficulty concentrating, trouble maintaining attention). On a 6-point scale (ranging from 1 = all of the time, to 6 = none of the time), participants indicate how often they experienced a specific cognitive problem during the previous week.

#### General health perception

As a general indicator of perceived health, participants are asked to rate their health in general on a 5-point scale (ranging from 1 = excellent to 5 = poor; general health item, SF-12 [83]). Additionally, participants indicate whether their health status interfered with their daily activities during the previous week on a 3-point scale (ranging from 1 = no problems, to 3 = unable to perform usual activities; usual activities item from EQ-5D [84]).

#### Perceived change in health

A one-item 7-point global rating of change scale [85] ranging from -3 (deteriorated) to + 3 (improved) is used to measure to what extent one’s health changed in the previous six months.

### Psychosocial health mechanisms

#### Causal beliefs about health complaints

Causal beliefs about health complaints are assessed in two different ways. First of all, participants are asked in an open format to write down the three most important causes of all health complaints experienced during the previous week (cf. IPQ-cause scale, [86]). Then they answer on a 6-point scale (from 1 = certainly not, to 5 = certainly and 6 = not applicable) whether they believe their health complaints are caused or worsened by a list of 11 environmental factors (busy road, chemical plant, contaminated land, overhead power transmission line, underground power transmission line, mobile phone base station, airport, intensive livestock farming, greenhouses, railroad track and wind turbine). This list of environmental risks is based on a scale used in local and national health monitors conducted by municipal health services and the national institute for public health and environment in the Netherlands [87]. The same list of environmental risk factors is used for all other questions regarding power lines. An ‘other environmental factors’ category is added to allow respondents to add other environmental factors they attribute their health complaints to.

#### Anxiety and depression

State anxiety and depression is measured using a Dutch translation [88] of the 14-item Hospital Anxiety and Depression Scale (HADS, [89]), using the timeframe of “last week”. The anxiety and depression subscales both consist of 7 items.

#### General and environmental health worries

General health concerns are measured using a Dutch translation [90] of the 4-item health concern scale of the RAND General Health Perceptions Questionnaire [91]. Respondents answer on a 5-point scale (1 = definitely true, 5 = definitely false) whether statements regarding health concerns corresponded to their own view. Concern about the effects of environmental factors on one’s own personal health is assessed with the Modern Health Worries scale [46] adapted to our list of 11 environmental factors. On a 5-point scale (1 = no concern, 5 = extreme concern), respondents indicate how concerned they are about the effects of the environmental factors on their own personal health.

### Perception of the environment

#### Health risk perception

Perceived health risks of environmental factors are measured in two different ways. On a general level, participants indicate on a 5-point scale whether they consider the environmental factors to be a health risk for residents living in the vicinity (ranging from 1 = certainly not, to 5 = certainly). On a personal level, participants indicate on the same 5-point scale whether they think they would get health complaints if they lived near these environmental factors.

#### Perceived proximity

As an indicator for perceived exposure to environmental risk factors, we ask participants to judge on a 5-point scale whether the environmental factors are close or far away from their home (1 = very close, 5 = very far, adapted from [92]). The scale will be recoded to reflect perceived proximity.

#### Perceived saliency of environmental health risk information

Since it is not possible to accurately record what information residents received about the health risks of high-voltage power lines, we have to rely on the perception of health risk information. Participants are asked to indicate how often during the previous three months they had heard or read about the health effects of power lines, amongst the other environmental factors (1 = never, 5 = very often).

#### Perceived change in living environment

We ask respondents to indicate whether they perceived any change in their living environment in the previous six months with regard to the list of 11 environmental factors in a no/yes format. In the case of a yes response, respondents could indicate what exactly had changed, and an ‘other’ category was added to allow for other environmental changes to be recorded. These items are included to check whether residents were aware of a change in their living environment regarding power lines after the Zuidring was put into operation. In addition, the results will give an impression of whether residents perceived any other salient environmental changes during the introduction of the Zuidring.

### Dispositional factors

#### Demographics

Gender, age, educational attainment, household income, years of residency at current address, marital and occupational status are recorded at the first measurement.

#### Neuroticism and lack of mastery

Two types of negative oriented personality traits are assessed. Neuroticism, defined as “a broad dimension of individual differences in the tendency to experience negative, distressing emotions and to possess associated behavioral and cognitive traits” ([60], p. 301), is measured with the 12-item Neuroticism subscale of the NEO Five-Factor Inventory (NEO-FFI, [93]). Respondents are asked to indicate on a 5-point Likert scale to what extent they agree with statements describing themselves. A perceived lack of mastery, which might be seen as a negative oriented personality characteristic indicative of a lack of psychological resources, is measured with the 5-item Mastery scale [94]. Statements regarding the lack of control over life are rated for applicability on a 5-point Likert scale.

### Statistical analyses

The first two research questions are about the effects of the introduction of a new power line on the primary outcome measures, i.e. non-specific health complaints and causal beliefs about these complaints. With an expected attrition rate of 30%, multilevel regression analyses are more appropriate than repeated measures ANOVA, because they allow all data available to be used instead of listwise deletion. Time will be entered as predictor and the pretests (T1 and T2) will be coded as a reference category to the posttests (T3 and T4). The cross-level interaction between time and distance to the Zuidring (0-500 m vs. 500-2000 m) will indicate whether the null hypothesis of no different growth between the QE and NEC group should be rejected. Exploratively, growth for the underground transmission line sample will be compared to the QE and NEC group using the same analyses. For demographics (e.g. sex, age, education etc.), confounding of the relationship between proximity to the Zuidring and our outcomes will be checked and corrected if necessary.

The complete conceptual framework (Figure 1) will be tested through the parallel process latent growth curve modeling approach [95]. First the growth trajectory of all likely mediators will be investigated and compared between the QE and NEC group. In the case of a good fit to the data, in a second step the models will be combined into one parallel process model and a confidence interval for the mediated effects will be calculated. Moderation of the meditational process will be checked through multigroup models for the dispositional factors.

## Discussion

We believe our study is an important addition to studies on the health effects of exposure to ELF-EMF from power lines. Only a few studies have investigated the psychosocial pathway from power lines to symptom reporting. This is in contrast to the numerous epidemiological and experimental studies investigating the associations and direct physical pathway between ELF-EMF emitted by power lines and health outcomes. For a full assessment of health effects, it is essential to study nocebo and attribution responses. Although these outcomes are subjective in nature and therefore may reflect a reporting bias, nocebo studies showed that negative expectations can alter brain activity and hormone levels associated with the experience of anxiety and pain [43,96]. This is in line with studies indicating differences between controls and self-diagnosed electro-hypersensitive patients in objective biological measurements such as cortical excitability [39], sympathetic skin response, brain responsiveness [97] and heart rate variability [98]. Therefore nocebo and attribution responses do not merely reflect a reporting bias. Under experimental conditions these types of psychosocial health effects were shown to be substantial.

The introduction of new high-voltage power lines in the Netherlands provides a unique opportunity to investigate health responses to power lines. Because the construction of a power line is planned (unlike many other environmental risk events), it enables prospective research of nocebo and attribution responses. Moreover, since exposure to this risk is spatially defined, the target group is easy to identify. Due to the fact that a relatively small part of the general population lives near a power line, studies without a specific sampling strategy will likely underestimate perceived risks. The strength of our study lies in the combination of this specific sampling strategy, combined with a broad environmental health risk approach, enabling comparisons with perception of other potential environmental hazards.

Another strong asset of our study is the quasi-experimental field design. Findings from nocebo and attribution studies conducted in laboratory settings may not fully extend beyond the laboratory. Perceived exposure is easier to manipulate in an isolated laboratory setting where no other environmental stressors are present. However, nocebo or attribution effects might partly be explained by demand characteristics, something experiments suffer more from than do observational studies. Another limitation of these controlled experiments is that symptom reporting is usually assessed right after or during perceived exposure, which does not provide information about how long-lived these effects are under everyday conditions. Our study provides the next step in nocebo research by determining the size of these effects under natural conditions.

To enhance further understanding of health responses to environmental risks, it is important to measure both non-specific health complaints as well as causal beliefs about these complaints. It is often suggested in the literature that residents may reattribute pre-existing health complaints after an environmental incident (i.e. [26-28,99]), but to our knowledge there is not much evidence from prospective field studies to support this claim. There are very few prospective studies on psychosocial responses to environmental health risks and often they do not assess causal beliefs. An exception is a study by Petrie and colleagues [67] of psychosocial health effects after environmental pesticide spraying. They found an effect of prior concerns about environmental hazards on the number of symptoms attributed to the spraying afterwards, while there was no such effect on the total number of symptoms reported afterwards. Because prior causal beliefs and perceived exposure to the spraying were not assessed, it remains unsure how the incident affected people’s perceptions. The longitudinal character of our study allows for relating change patterns in environmental attribution to change patterns in symptom reporting, before and after the introduction of a potential environmental risk. This provides insight into the exact role of causal beliefs in the development of complaints during a potential environmental risk event.

Our chosen approach also has its limitations. As it is not possible to randomly assign residents to live near or farther away from a power line, strong external validity comes with a loss in internal validity. We augmented the standard pretest-posttest design by having two pre- and two posttests and a non-equivalent control group, to offer more protection against threats to internal validity, such as history, maturation and testing. We do not know what determined whether residents live near or farther away from the new line. One factor (to live in a rural or urban environment) was controlled for by means of a stratified sampling strategy. Other potential confounders (i.e. demographic variables) are measured to control for in the statistical analyses, if necessary. One factor we cannot control for is the potential influence of ELF-EMF. Although actual exposure might have an effect on health, it should be mentioned that exposure to ELF-EMF from power lines rapidly decreases with distance [100]. Upward of 55 meters from the heart of the Zuidring, the expected average magnetic field strength is already below 0.4 microTesla [101], which is suggested as a cut-off value for the higher relative risk of childhood leukemia [5]. Only a few included households reside this close to the Zuidring.

There are also limits to the generalizability of our results. First of all, the present design concerns a case study of one new power line route in one country. Since policy regarding the introduction of new high-voltage power lines and concern about the health effects differs between countries (see [102]), the results may not apply to other societies. In addition, there might be differences between various new power lines routes with regard to opposition and media attention, translating to different psychosocial responses on a more local level. Second, although we think our conceptual framework might apply to the introduction of other potential environmental risk objects as well (e.g. mobile phone base stations, wind turbines, chemical plant, etc.), the results of our study do not automatically extend to these other risks. Cross-validation of our framework with other risk factors and other high-voltage power line routes is therefore warranted.

To summarize, the present study protocol described the design of a case study of potential health responses after the introduction of a new high-voltage power line route in the Netherlands. We defined health responses as an increase in non-specific health complaints and attribution of these complaints to a power line, triggered by the introduction of a new power line in a residential area. We presented a conceptual framework outlining nocebo and attributional mechanisms potentially explaining health responses, and we will test this framework for the present case. For researchers in the fields of psychosomatic medicine, environmental health and environmental psychology, the results may provide theoretical insight into psychosocial mechanisms operating during the introduction of an environmental health risk. The results of this study can provide stakeholders focal points for minimizing adverse health responses to new high-voltage power lines.

## Abbreviations

ELF-EMF: Extremely low frequent electromagnetic fields; EMF: Electromagnetic fields; NOP: Negative oriented personality; QE: Quasi-experimental; NEC: Non-equivalent control; 4DSQ: Four-dimensional symptom questionnaire; MOS: Medical outcomes study; SF-12: 12-item short form survey; EQ-5D: European quality of life index version 5D; IPQ: Illness perception questionnaire; HADS: Hospital Anxiety and Depression Scale; GHPQ: RAND General Health Perceptions Questionnaire; MHW: Modern Health Worries Scale; NEO-FFI: NEO Five-Factor Inventory.

## Competing interests

The authors declare that they have no competing interests.

## Authors’ contributions

All authors contributed to the design of the study. JP wrote the manuscript, which was commented on by all authors. DT is the principal investigator and LC, FW and TS were co-applicants of the proposal. All authors read and approved the final manuscript.

## Pre-publication history

The pre-publication history for this paper can be accessed here:

http://www.biomedcentral.com/1471-2458/14/237/prepub
